# Role of Genomic, Economic, and Demographic Disparities in Mpox Epidemic in Africa: A Retrospective Cross-Country Analysis

**DOI:** 10.3390/microorganisms13112531

**Published:** 2025-11-05

**Authors:** Blondy Kayembe-Mulumba, Anderson Kouabenan N’gattia, Marie Roseline Darnycka Belizaire, Thomas D’Aquin Koyazegbe, Marcel Mbeko Simaleko, Yap Boum, Pierre Somsé

**Affiliations:** 1 Bordeaux Population Health Research Center, BPH, INSERM, U1219, University of Bordeaux, F-33000 Bordeaux, France; 2 National Institute of Public Hygiene, Ministry of Health, 01 BPV Abidjan, Côte d’Ivoire; 3 World Health Organization, Country Office of the Central African Republic, BP 1416 Bangui, Central African Republic; 4 School of Medicine, University of Alcalá, 28801 Madrid, Spain; 5 Faculty of Health Sciences, University of Bangui, BP 1426 Bangui, Central African Republic; 6 Policy, Strategy and Cooperation Monitoring Division, Ministry of Health and Population, BP 883 Bangui, Central African Republic; 7 Pasteur Institute of Bangui, BP 923 Bangui, Central African Republic; 8 Office of the Minister, Ministry of Health and Population, BP 883 Bangui, Central African Republic

**Keywords:** mpox epidemic, viral clade, GDP per capita, population density, global health, Africa

## Abstract

To investigate the role of epidemic predictors in the mpox outbreak in Africa. This was a retrospective analysis of national-level mpox surveillance data from 20 mpox-affected African countries from January through December 2024. Predictors included viral clades, gross domestic product (GDP) per capita, and population density. A negative binomial regression model estimated the incidence rates ratio (IRR) [95% confidence interval] for mpox incidence and mortality. Random forest models assessed the influence of each predictor in the epidemic dynamic. Clade II was associated with lower mpox incidence (IRR = 0.15 [0.02–0.97]) and mortality (IRR = 0.09 [0.01–1.72]) compared to Clade I. GDP per capita was associated with a 95% reduction in cases count per US $1000 (IRR = 0.05 [0.38–0.74]). Population density was not significantly associated with mpox incidence or mortality. Random forest analysis confirmed GDP per capita as the strongest predictor of mpox burden. The 2024 mpox epidemic highlights how countries with low GDP per capita and Clade I face greater outbreak burdens. Strengthening health systems and addressing poverty as a key social determinant of health through a multisectoral approach are essential to ensure equitable outbreak prevention, control, and long-term resilience.

## 1. Introduction

Mpox, formerly known as monkeypox, emerged in the 1970s in Central and Western Africa, following smallpox eradication [1]. The disease, caused by the monkeypox virus, presents with symptoms such as fever, lymphadenopathy, and a distinctive rash [2]. Although generally self-limiting, mpox can lead to severe complications, especially in children, immunocompromised individuals, and pregnant women [2]. The disease was first identified in humans in 1970 in the Democratic Republic of the Congo (DRC), and for decades remained largely endemic to Central and West Africa. However, in recent years, mpox has demonstrated an alarming capacity for international spread [3,4]. By 2022, cases had been reported in over 100 countries, including many without previous history of mpox transmission. According to the World Health Organization (WHO), more than 85,000 confirmed cases and over 100 deaths were recorded globally by the end of 2023, with a case fatality rate (CFR) ranging from 0.1% to 10%, depending on clade and healthcare access [4]. Mpox is primarily transmitted through close physical contact with infected skin lesions, body fluids, or respiratory droplets. Human-to-human transmission can also occur via contaminated materials (fomites), and in some instances, zoonotic transmission from animals to humans has been documented [1]. Key risk groups include healthcare workers, men who have sex with men (MSM), household contacts of confirmed cases, and individuals living in endemic areas with close contact with wildlife [5]. Vaccination with smallpox vaccines (e.g., JYNNEOS or ACAM2000) provides cross-protection against mpox and has been used in targeted immunization strategies [6,7]. Other preventive measures include isolation of cases, use of personal protective equipment (PPE) by healthcare workers, contact tracing, and public awareness campaigns to reduce risky behaviors and improve hygiene practices.

Historically endemic to Central and Western Africa, mpox has recently demonstrated a concerning potential for global spread occurring for the first time in several non-endemic countries in 2022 [3,4]. In August 2024, WHO declared an upsurge of mpox cases in DRC and neighboring African countries as a Public Health Emergency of International Concern, just two years after that of 2022 [8]. This declaration underscores the escalating threat mpox poses, not only to affected regions but also to global health security, as it challenges healthcare infrastructures and necessitates coordinated international responses [9].

Understanding the factors that facilitate the spread of mpox, particularly in Africa, is crucial for developing effective containment and prevention strategies. Several potential risk factors have been identified in the spread of epidemics, including viral differences, socioeconomic indicators such as gross domestic product (GDP) per capita, and demographic factors like population density [10,11,12]. The identification of a novel viral clade with enhanced transmissibility in the DRC has raised concerns about the evolutionary dynamics of mpox and its implications for outbreak control [13]. Socioeconomic disparities, as reflected by GDP per capita, may affect the capacity of health systems to respond effectively, influencing vaccine distribution and public health outreach in resource-limited settings [14]. In addition, high population densities, particularly in urban areas, can facilitate human-to-human transmission by increasing the likelihood of close contact events [15]. Previous studies have typically focused on single aspects of mpox transmission or were limited by small sample sizes and short observation periods, which constrains their ability to capture the multifaceted interplay of these determinants [16]. Moreover, many investigations were region-specific and did not account for the broader socio-economic context that influences epidemic trajectories [17]. This knowledge gap highlights an urgent need for comprehensive studies that integrate virological, socioeconomic, and demographic factors to elucidate the underlying mechanisms driving mpox spread.

Thus, the overarching objective of this study was to identify factors influencing the 2024 mpox epidemic spread in Africa. More specifically, the study aimed to analyze the roles of viral clade variations, GDP per capita, and population density as drivers of the 2024 mpox epidemic spread across African countries.

## 2. Materials and Methods

### 2.1. Study Design and Population

This study employed a retrospective cross-country design to investigate the determinants of the 2024 mpox epidemic in Africa. Data were collected from 20 affected African countries, with aggregation at the national level. The study period encompassed all reported cases and deaths from January to December 2024, ensuring a comprehensive analysis of epidemic trends. The selection of countries was based on the report of at least one confirmed mpox new case from national health authorities, acknowledged by WHO.

### 2.2. Data Sources

Surveillance data on mpox cases and deaths were obtained from WHO’s global dataset (https://worldhealthorg.shinyapps.io/mpx_global/, accessed on 19 January 2025). These data are provided by national Ministries of Health (MoH) and compiled by WHO after quality assessment and validation. Following WHO’s mpox surveillance and reporting guidelines [18], a national health authorities report confirmed mpox cases and deaths on a weekly basis, aggregated at the national level. Viral clade distribution was identified based on genomic sequencing data provided by national or regional reference laboratories [18]. Socioeconomic and demographic data, as of 2023, including GDP per capita and population density, were extracted from the World Bank database (https://data.worldbank.org, accessed on 27 February 2025) [19]. Authors had no access to information that could identify individual participants during or after data collection.

### 2.3. Study Variables

Our final dataset included dependent and predictor variables. Dependent variables comprised weekly mpox cases (defined as the total number of newly confirmed mpox cases within an epidemiological week) and deaths (defined as the total number of confirmed mpox-related deaths within an epidemiological week). We calculated the mpox weekly case fatality rate (CFR, mortality attributed to mpox) as the total new deaths by the total new cases within an epidemiological week.

Predictor variables included viral clades (classified into Clade I, Clade Ia, Clade Ib, Clade II, and Undefined, based on genomic sequencing), GDP per capita (current United States Dollar [US $], reflecting the economic status of the country), population density (inhabitant per km^2^, used as a proxy for transmission potential in densely populated areas), and African regions (Central, Eastern, Western, Southern, and Northern). Other variables related to the country’s unique ISO identifier and the date of the epidemiological week.

### 2.4. Analytical Strategy

Descriptive statistics were used to summarize the characteristics of the affected countries, including median and interquartile ranges (IQR) for continuous variables and proportions for categorical variables. Spatial and temporal trends in mpox incidence and mortality were visualized using epidemic curves and distribution graphs. We conducted a bivariate analysis (Pearson’s rho) to evaluate the correlation of quantitative predictors with mpox metrics.

To assess the association between predictor variables and mpox outcomes, a multivariate negative binomial regression (NBR) model was employed—over Poisson regression—due to the overdispersed-count nature of case and death data. We analyzed incidence and mortality in separate models. Incidence rate ratios (IRRs) with 95% confidence intervals (CIs) were estimated to quantify the effect of GDP per capita, population density, and viral clades on mpox incidence and mortality. Viral clades were grouped into clades I and II—the undefined category was imputed to the mode, which was clade I—to handle the small number of countries in subclade categories. The model assumptions and conditions were thoroughly checked. Overdispersion was assessed using the likelihood ratio test comparing Poisson and NBR models, confirming that the negative binomial distribution was appropriate. Multicollinearity among predictor variables was evaluated using the variance inflation factor, ensuring that no variables exhibited problematic collinearity. The model’s goodness-of-fit was assessed using Pearson residual plots and the Akaike Information Criterion, which indicated an adequate fit. Additionally, we employed a random forest model to evaluate the relative importance of predictors in determining mpox spread and fatality. This approach provided a non-parametric alternative, enabling assessment of predictor influence without making strict distributional assumptions.

All analyses were conducted using *R* software, version 4.4.1 (R foundation for statistical computing, http://www.r-project.org), with statistical threshold set at 5%. This study utilized publicly available, de-identified data. Therefore, formal ethical approval was not required. However, all data sources were acknowledged, and analyses were conducted in accordance with ethical guidelines for secondary data use.

## 3. Results

### 3.1. Characteristics of Analyzed Countries

Twenty African countries met the inclusion criterion by reporting at least one new case of mpox in 2024. The distribution of mpox cases across these affected countries highlights significant heterogeneity in epidemic burden (Supplementary Table S1). The Democratic Republic of the Congo (DRC) reported the highest cumulative case count (9513 confirmed cases, 65.29% of total cases), followed by Burundi (2946 confirmed cases, 20.22%) and Uganda (1487 confirmed cases, 10.21%), as depicted in Supplementary Figure S1. The death count varied considerably, with the highest observed in DRC (43 deaths) and Uganda (10 deaths). However, Cameroon featured the highest CFR (22%), followed by South Africa (12%). Notably, while some countries with high case burdens reported minimal deaths, others with fewer cases exhibited relatively high CFRs. Half of the countries were affected by Clade I and its subtypes (Ia and Ib), particularly in Central and Eastern Africa. Countries with higher population densities, such as Rwanda and Burundi, exhibited relatively high case burdens (Supplementary Table S1).

The median number of reporting weeks was 10.0 (IQR: 2.5–22.5), reflecting variability in the duration of epidemic activity among countries (Table 1). The median number of confirmed cases was 24.5 (IQR: 3.0–98.5), with considerable variation from as few as one case (Mauritius) to over 9000 cases (DRC). In terms of viral clades, 50% of countries reported Clade I, 35% Clade II, and 15% had undefined clades. The median population density was 72.1 inhabitant/km^2^ (IQR: 43.0–171.8), and the median GDP per capita was US $1844.6 (IQR: 1006.3–2504.4).

**Figure 1:**
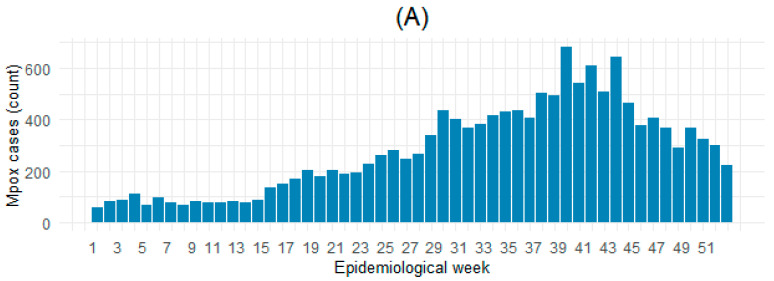

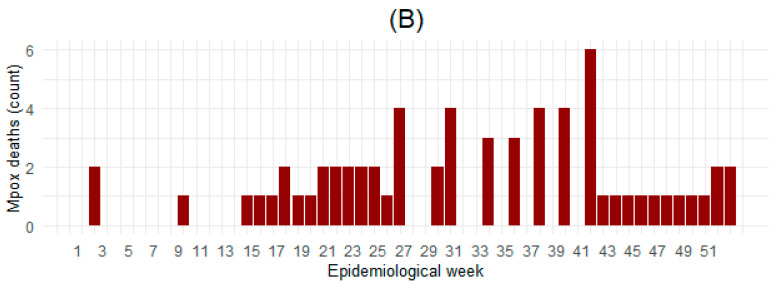
Mpox epidemic curves. Plots show weekly mpox (**A**) cases and (**B**) deaths in African countries (n = 20) in 2024.

**Figure 2:**
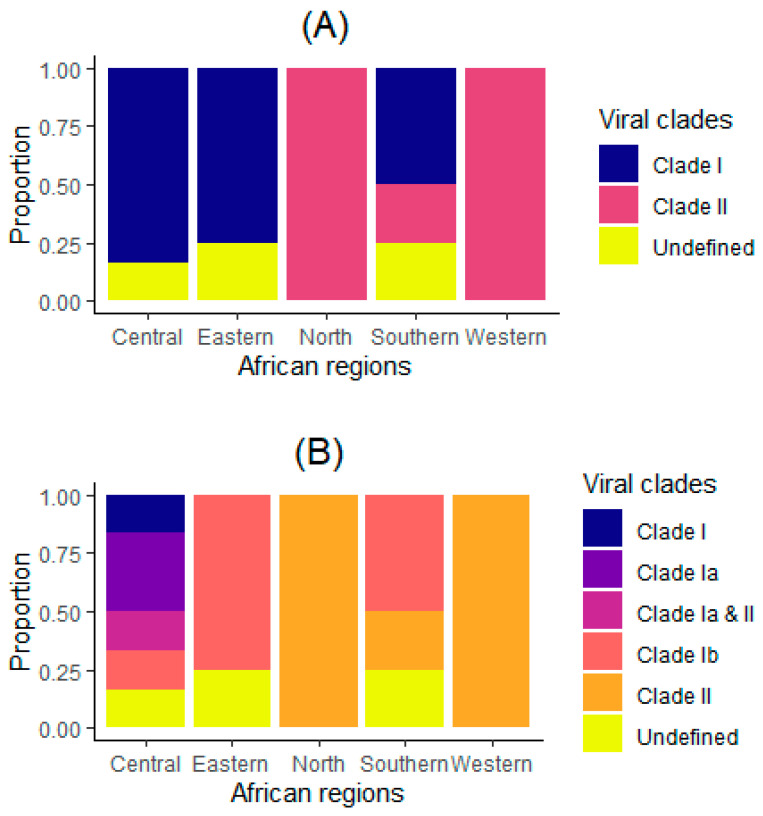
Distribution of viral clades by African regions. Regional distribution of mpox viral strains in (**A**) three and (**B**) six clades in African countries (n = 20) in 2024.

**Figure 3:**
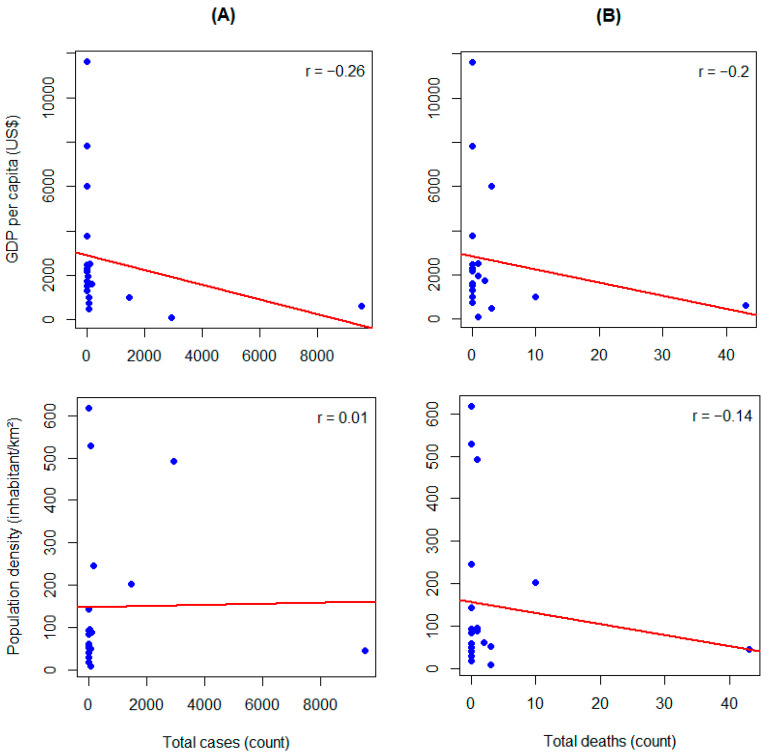
Plots of correlation between predictors and mpox epidemic. Correlation of total mpox (**A**) cases and (**B**) deaths with GDP per capita (first row) and population density (second row) in African countries (n = 20) in 2024. Red solid line indicates correlation direction. Blue dots represent countries. Abbreviations: GDP, gross domestic product; r, rho as Pearson’s correlation coefficient; US $, United States dollar.

The epidemic curve reveals a sharp rise in cases at the onset of the outbreak, followed by fluctuations in incidence over time (Figure 1A). Transmission peak occurred around the 40th week. Mortality trends loosely mirrored case incidence (Figure 1B). The cumulative incidence and mortality trends demonstrate continuous outbreak expansion, with a steady evolution of CFR over time (Supplementary Figure S2). Clade I and its subtypes were predominantly observed in Central and Eastern Africa, while Clade II was more common in Western and Southern Africa, with the presence of mixed subclades in some regions, especially in the Central and Southern regions (Figure 2). We found that mpox incidence was inversely weakly associated with GDP per capita (r = −0.26), while no correlation was found with regard to population density (r = 0.01) (Figure 3A). Figure 3B portrays a weak inverse association of mpox mortality with both GDP per capita (r = −0.2) and population density (r = −0.14).

### 3.2. Identification of Factors Associated with the Mpox Epidemic

**Figure 4:**
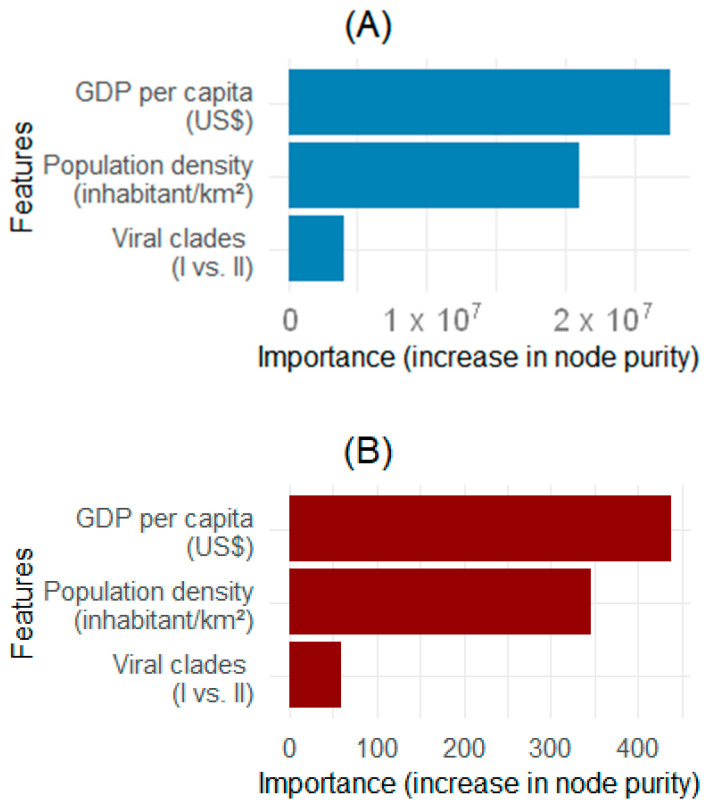
Predictors’ influence on mpox epidemic. Plots from random forest models showing influence of predictors on mpox (**A**) cases and (**B**) deaths counts during the 2024 outbreak in African countries (n = 20). Abbreviations: GDP, gross domestic product; US $, United States dollar.

The multivariate NBR model (Table 2) revealed that a US $1000 increase in GDP per capita was significantly associated with a 95% decrease in mpox cases (IRR = 0.053, *p* < 0.001). Clade II was significantly associated with 85% lower cases compared to Clade I (IRR = 0.147, *p* = 0.047). Clade II was associated with a 91% lower mortality rate than Clade I (IRR = 0.093, *p* = 0.110), albeit statistical significance was not reached. Random forest analysis unveiled GDP per capita as the strongest predictor of mpox incidence (over 25 million increase in node purity), followed by the population density (over 20 million increase in node purity) (Figure 4A). Consistently, GDP per capita and population density had the highest but moderate influence on mpox mortality, with nearly 350 and 250 increase in node purity, respectively (Figure 4B).

## 4. Discussion

Our study identifies viral clade variation and GDP per capita as significant determinants of the 2024 mpox epidemic spread in Africa. The findings indicate that Clade I is associated with higher incidence and mortality rates compared to Clade II. Additionally, higher GDP per capita is strongly associated with lower mpox incidence, while population density does not show a significant direct association.

To the best of our knowledge, this is the first study that investigated the association of virological, socio-economic, and demographic factors with mpox epidemic at cross-country level. Therefore, we lacked existing empirical data at cross-country level for direct comparison with our results. Nevertheless, our findings align with previous research that supported the differential virulence and transmissibility of mpox viral clades from in-country data. Clade I has historically been associated with more severe disease outcomes and higher mortality rates than Clade II [20]. This distinction is critical for epidemic response strategies, as it suggests that countries with Clade I circulation may require enhanced surveillance and more aggressive containment measures. Previous studies have highlighted the importance of genomic surveillance in monitoring viral evolution and assessing potential shifts in transmissibility and virulence [20,21], reinforcing the need for continuous genomic monitoring of mpox cases. Furthermore, our study underscores the necessity for region-specific mpox response strategies. The significantly higher incidence and mortality associated with Clade I, which is region-specific, may necessitate coordinated and targeted interventions, such as intensified surveillance, rapid case detection, and early therapeutic interventions in the Central, Eastern, and Western regions.

Regarding socioeconomic factors, our study corroborates the role of GDP per capita, as a proxy of national income and health system strength, in influencing epidemic outcomes. Previous research on emerging infectious diseases has highlighted the inverse relationship between national income and health emergency response. They reported the association of higher GDP per capita with stronger healthcare infrastructure, better disease surveillance, and increased access to preventive measures such as vaccination and public health interventions [10,22,23]. Similarly, a study emphasizes that resource-limited settings struggle to contain outbreaks due to inadequate medical resources and limited healthcare accessibility, further supporting our findings [16]. In our study, the strong inverse relationship between GDP per capita and mpox incidence suggests that economic factors play a critical role in epidemic preparedness or containment. It can be assumed that countries with higher national income had stronger health systems to control mpox spread.

Countries with high GDP such as Thailand, Sweden, Germany, Belgium, the United Kingdom, Canada, India, Oman, Pakistan, and the United States have reported cases linked to travel from African countries affected by Clade I, yet have not experienced a surge. This is likely due to robust health systems and vaccine availability. Consequently, policymakers should prioritize investments in healthcare infrastructure, particularly in lower-income countries, to improve outbreak preparedness and response capacity [11,24]. Strengthening health systems through increased funding, improved healthcare workforce training, and expanded access to mpox vaccines and therapeutics can mitigate the impact of future outbreaks [14,25]. Vaccination must be prioritized as a critical intervention, especially in low- and middle-income countries with weaker health systems, where implementing standard IPC (infection prevention and control) and surveillance measures is more challenging. This is particularly true given mpox sexual transmission route, which complicates contact tracing and highlights the need for targeted vaccination strategies to control its spread. Moreover, investing in long-term sustainable development sectors (e.g., education, employment, etc.) may be part of health system strengthening and preparedness for future mpox epidemics. Population density has been widely recognized as a determinant of infectious disease spread [12,15,26], yet our study did not find a statistically significant association between population density and mpox incidence or mortality. This may be due to the predominant transmission mode of mpox, which requires close physical contact rather than airborne spread, unlike other viral infections such as influenza or SARS-CoV-2 [27,28]. Additionally, we used data aggregated at national level, not allowing considering population density in affected areas within individual countries, as population density is not homogeneous within a country. Furthermore, our results are consistent with studies suggesting that localized epidemiological factors, such as social behaviors, healthcare access, and cultural practices, may play a more substantial role in transmission dynamics than population density alone [29,30]. Nonetheless, although population density did not emerge as a significant predictor, its potential indirect role should not be overlooked. High-density settings, particularly in urban areas, may facilitate mpox transmission through increased skin-to-skin interactions. Public health interventions, such as targeted community education, contact tracing, and isolation measures, remain crucial for controlling transmission in densely populated areas. Previous research has emphasized the importance of social behavior modifications in controlling infectious diseases [12,17], suggesting that public health messaging tailored to specific populations can enhance compliance with preventive measures. The confirmation of clade I sexual transmission by Kibungu and colleagues only shapes this argument [21].

This study contributes to the growing body of evidence on mpox epidemiology by integrating virological, socioeconomic, and demographic factors into a comprehensive analytical framework. Socioeconomic factors measurement (2023) preceded the mpox outbreak (2024), which enhances causal relationship while minimizing reverse causation bias. The use of multivariate NBR models ensures robust estimation of associations while accounting for data overdispersion. Furthermore, the incorporation of Random Forest models provides a complementary, non-parametric assessment of variable importance, reinforcing our primary regression-based results.

Despite its strengths, our study has some limitations. First, the cross-country design precludes causal inference at the individual level (ecological fallacy). The aggregated nature of the data may mask within-country heterogeneity in mpox transmission dynamics. Second, although we accounted for major predictors, unmeasured confounders such as healthcare access, vaccination coverage, and behavioral factors may influence mpox spread and severity. Additionally, the limited number of countries affected by subclades Ia (two) and Ib (six) precluded the analysis of their individual influence. Future research should prioritize such analyses to clarify their respective influence in mpox epidemic dynamics. Third, the use of national-level GDP per capita as a socioeconomic indicator does not capture disparities within countries, which could affect healthcare accessibility and epidemic outcomes. Lastly, our study relies on surveillance data, which may be subject to reporting biases, particularly in regions with limited diagnostic capacity or underreporting tendencies. Future research should explore individual-level risk factors—such as age, sex, and autonomy—and incorporate seroprevalence data to refine our understanding of mpox transmission patterns.

## 5. Conclusions

The mpox epidemic in Africa reveals a stark intersection between viral strain dynamics and economic inequity. Our findings show a significant association of Clade I and lower GDP per capita with mpox spread, underscoring that countries with limited economic resources bear a disproportionate burden of the outbreak. This highlights the urgent need for targeted investments in surveillance, vaccination, and health system strengthening in lower-income settings. Beyond the health sector, a comprehensive and multisectoral approach is essential to address poverty as a fundamental social determinant of health and reinforce resilience across sectors to effectively prevent and control future outbreaks.

## Figures and Tables

**Table 1:** Descriptive characteristics of the African countries (n = 20) affected by mpox outbreak in 2024.

Characteristics	No. (%)	Median (IQR) [Min; Max]
Reporting weeks (count) ^a^	278	10.0 (2.5; 22.5) [1.0; 46.0]
Cases (count) ^a^	14,570	24.5 (3.0; 98.5) [1.0; 9513.0]
Deaths (count) ^a^	64	0.0 (0.0; 1.0) [0.0; 43.0]
GDP per capita (US $)	20	1844.6 (1006.3; 2504.4) [109.0; 11,623.0]
Population density (inhabitant/km^2^)	20	72.1 (43.0; 171.8) [8.3; 618.1]
Viral clades (n = 20)		
Clade I	10 (50)	NA
Clade II	7 (35)	NA
Undefined	3 (15)	NA
Viral subclades (n = 20)		
Clade I	1 (5)	NA
Clade Ia	2 (10)	NA
Clade Ia & II	1 (5)	NA
Clade Ib	6 (30)	NA
Clade II	7 (35)	NA
Undefined	3 (15)	NA
Regions (n = 20)		
Central	6 (30)	NA
Eastern	4 (20)	NA
North	1 (5)	NA
Southern	4 (20)	NA
Western	5 (25)	NA

Abbreviations: GDP, gross domestic product; IQR, interquartile range; n, number of analyzed countries; NA, not applicable; US $, United States dollar; ^a^ Cumulative count from all the countries.

**Table 2:** Incidence rate ratios (IRR) of mpox epidemic determinants in African countries (n = 20). Results from the multivariate negative binomial regression model (count model).

Epidemic Determinants	Mpox Cases		Mpox Deaths	
	IRR [95% CI]	*p*-Value	IRR [95% CI]	*p*-Value
GDP per capita (per US $1000 increase)	0.53 [0.38–0.74]	**<0.001**	0.93 [0.53–1.65]	0.813
Population density (per 100-unit increase)	1.00 [0.59–1.70]	0.989	0.55 [0.16–1.17]	0.119
Viral clade				
Clade I (reference)	1	**0.047**	1	0.110
Clade II	0.15 [0.02–0.97]		0.93 [0.01–1.72]	

Abbreviations: IRR, incidence rate ratio; CI, confidence interval; GDP, gross domestic product; US $, United States dollar. Statistically significant *p*-values are indicated in bold.

## Data Availability

All the data are publicly available in accessible repositories of the WHO (https://worldhealthorg.shinyapps.io/mpx_global/, accessed on 19 January 2025) and the World Bank (https://data.worldbank.org, accessed on 27 February 2025). The metadata generated in this manuscript is provided in Supplementary Table S1.

## Supplementary Materials

The following supporting information can be downloaded at: https://www.mdpi.com/article/10.3390/microorganisms13112531/s1, Figure S1: Mpox epidemic curves. Graphs show weekly mpox confirmed cases by country (n = 20) during the 2024 outbreak in Africa; Figure S2: Mpox epidemic metrics trend. Graphs show cumulative confirmed cases (dotted blue line), deaths (circle red line) and case fatality ratio (dashed black line) in the 20 countries affected by the 2024 mpox outbreak in Africa; Table S1: Profile description of individual countries (n = 20).

## Abbreviations

The following abbreviations are used in this manuscript:

GDP	Gross domestic product
CFR	Case fatality rate
MoH	Ministry of Health
DRC	Democratic Republic of Congo
WHO	World Health Organization
NBR	Negative binomial regression
IRR	Incidence rate ratio
