# Supplementary material for: Role of Genomic, Economic, and Demographic Disparities in Mpox Epidemic in Africa: A Retrospective Cross-Country Analysis
Source: Microorganisms. 2025 Nov 5;13(11):2531. doi: 10.3390/microorganisms13112531 (PMC12654172; doi:10.3390/microorganisms13112531)
Supplement: Supplementary file 1 [file microorganisms-13-02531-s001.zip › Figure_S1.pdf]

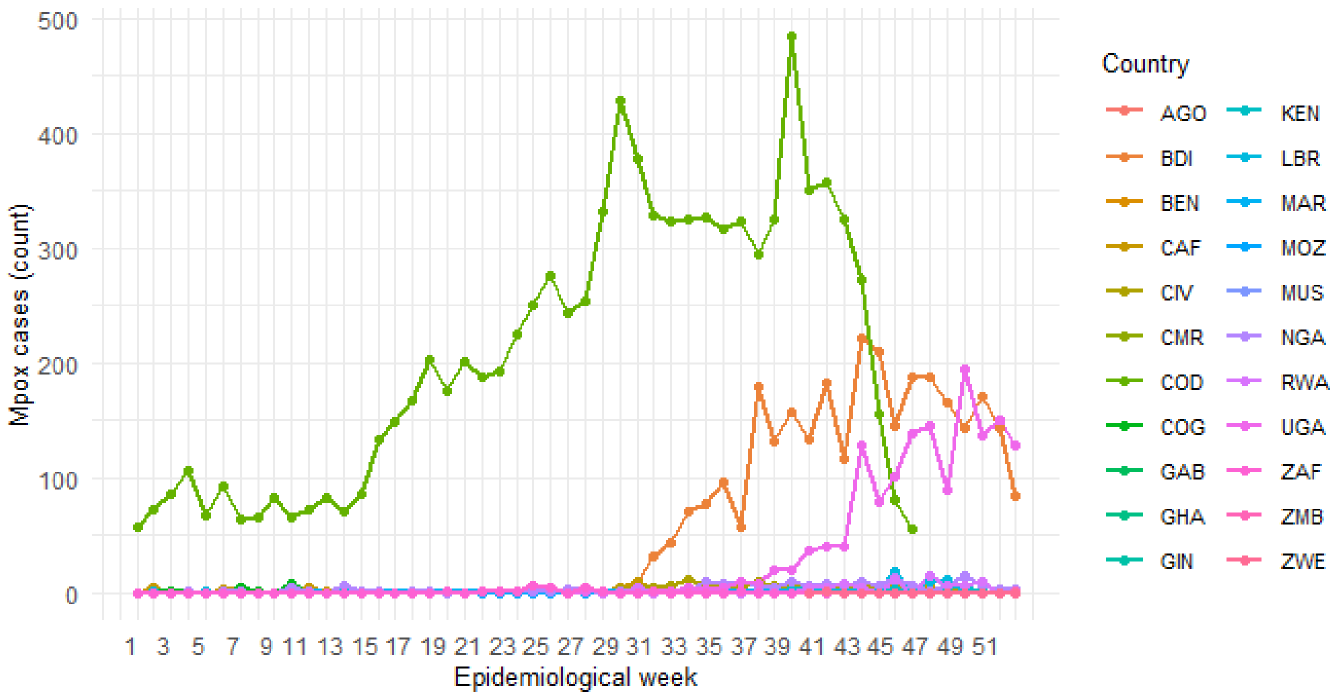

Figure S1. Mpox epidemic curves. Graphs show weekly mpox confirmed cases by country (n = 20) during the 2024 outbreak in Africa.
