# Supplementary material for: Role of Genomic, Economic, and Demographic Disparities in Mpox Epidemic in Africa: A Retrospective Cross-Country Analysis
Source: Microorganisms. 2025 Nov 5;13(11):2531. doi: 10.3390/microorganisms13112531 (PMC12654172; doi:10.3390/microorganisms13112531)
Supplement: Supplementary file 1 [file microorganisms-13-02531-s001.zip › Figure_S2.pdf]

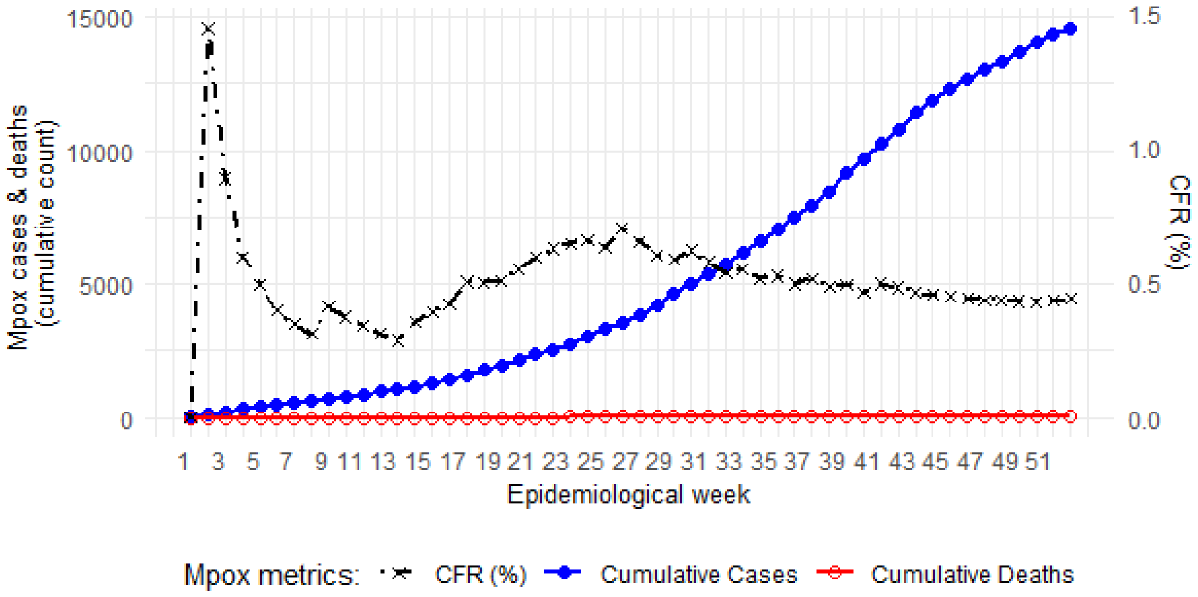

Figure S2. Mpox epidemic metrics trend. Graphs show cumulative confirmed cases (dotted blue line), deaths (circle red line) and case fatality ratio (dashed black line) in the 20 countries affected by the 2024 mpox outbreak in Africa.
