# Supplementary material for: Role of Genomic, Economic, and Demographic Disparities in Mpox Epidemic in Africa: A Retrospective Cross-Country Analysis
Source: Microorganisms. 2025 Nov 5;13(11):2531. doi: 10.3390/microorganisms13112531 (PMC12654172; doi:10.3390/microorganisms13112531)
Supplement: Supplementary file 1 [file microorganisms-13-02531-s001.zip › Table_S1.pdf]

Table S1. Profile description of individual countries (n = 20).

Countries	Code (iso3)	African regions	Reporting weeks (count)	Cases (count)	Cases (%)[a]	Weekly mean cases count	Deaths count	CFR (%)[b]	Viral clades	Viral subclades	GDP per capita (US$)	PD (inhab/km²)
Angola	AGO	Southern	4	4	0.03	1	0	0.00	Undefined	Undefined	2308.16	29.5
Burundi	BDI	Central	23	2946	20.22	128	1	0.03	Clade I	Clade Ib	109.01	491.8
CAR	CAF	Central	35	90	0.62	3	3	3.33	Clade I	Clade Ia	495.98	8.3
Côte d'Ivoire	CIV	Western	22	107	0.73	5	1	0.93	Clade II	Clade II	2530.85	87.7
Cameroon	CMR	Central	5	9	0.06	2	2	22.22	Clade I	Clade Ia & II	1736.86	59.7
DRC	COD	Central	46	9513	65.29	207	43	0.45	Clade I	Clade I	627.50	45.1
Congo	COG	Central	10	24	0.16	2	0	0.00	Clade I	Clade Ia	2477.98	18.1
Gabon	GAB	Central	2	2	0.01	1	0	0.00	Undefined	Undefined	7802.84	92.8
Ghana	GHA	Western	5	5	0.03	1	0	0.00	Clade II	Clade II	2260.29	141.7
Guinea	GIN	Western	2	2	0.01	1	0	0.00	Clade II	Clade II	1541.04	58.6
Kenya	KEN	Eastern	14	31	0.21	2	1	3.23	Clade I	Clade Ib	1952.30	95
Liberia	LBR	Western	19	63	0.43	3	0	0.00	Clade II	Clade II	771.89	49.3
Morocco	MAR	North	3	3	0.02	1	0	0.00	Clade II	Clade II	3771.45	84.4
Mauritius	MUS	Eastern	1	1	0.01	1	0	0.00	Undefined	Undefined	11623.04	618.1
Nigeria	NGA	Western	39	179	1.23	5	0	0.00	Clade II	Clade II	1596.64	246.7
Rwanda	RWA	Eastern	11	74	0.51	7	0	0.00	Clade I	Clade Ib	1010.27	529.7
Uganda	UGA	Eastern	24	1487	10.21	62	10	0.67	Clade I	Clade Ib	1002.31	201.9
South Africa	ZAF	Southern	10	25	0.17	3	3	12.00	Clade II	Clade II	6022.54	51.8
Zambia	ZMB	Southern	2	3	0.02	2	0	0.00	Clade I	Clade Ib	1330.73	27.5
Zimbabwe	ZWE	Southern	1	2	0.01	2	0	0.00	Clade I	Clade Ib	2156.03	40.9
**TOTAL**	**-**	**-**	**278**	**14570**	**100.00**	**52**	**64**	**0.44**	**-**	**-**	**-**	**-**

Abbreviations: CFR, case fatality rate; GDP· gross domestic product; US$· United States dollar; PD· population density; inhab/km²· inhabitant per square kilometers; CAR· Central African Republic; DRC· Democratic Republic of Congo. [a] Proportion of confirmed cases by country, calculated as the cases count in a given country divided by the total cases count (14570), times 100. [b] CFR is calculated as the number of mpox-related deaths (deaths count) in a given country divided by the number of confirmed cases (cases count) in the same country, times 100.
